# Dual-Color Fluorescence Imaging of EpCAM and EGFR in Breast Cancer Cells with a Bull’s Eye-Type Plasmonic Chip

**DOI:** 10.3390/s17122942

**Published:** 2017-12-19

**Authors:** Shota Izumi, Shohei Yamamura, Naoko Hayashi, Mana Toma, Keiko Tawa

**Affiliations:** 1 School of Science and Technology, Kwansei Gakuin University, 2-1 Gakuen, Sanda, Hyōgo 669-1337, Japan; S.izumi@kwansei.ac.jp (S.I.); m.toma@kwansei.ac.jp (M.T.); 2 Health Research Institute, National Institute of Advanced Industrial Science and Technology (AIST), 2217-14, Hayashi-cho, Takamatsu, Kagawa 761-0395, Japan; yamamura-s@aist.go.jp (S.Y.); nkhayashi884@gmail.com (N.H.)

**Keywords:** surface plasmon, fluorescence microscopic observation, breast cancer cell, membrane protein

## Abstract

Surface plasmon field-enhanced fluorescence microscopic observation of a live breast cancer cell was performed with a plasmonic chip. Two cell lines, MDA-MB-231 and Michigan Cancer Foundation-7 (MCF-7), were selected as breast cancer cells, with two kinds of membrane protein, epithelial cell adhesion molecule (EpCAM) and epidermal growth factor receptor (EGFR), observed in both cells. The membrane proteins are surface markers used to differentiate and classify breast cancer cells. EGFR and EpCAM were detected with Alexa Fluor^®^ 488-labeled anti-EGFR antibody (488-EGFR) and allophycocyanin (APC)-labeled anti-EpCAM antibody (APC-EpCAM), respectively. In MDA-MB231 cells, three-fold plus or minus one and seven-fold plus or minus two brighter fluorescence of 488-EGFR were observed on the 480-nm pitch and the 400-nm pitch compared with that on a glass slide. Results show the 400-nm pitch is useful. Dual-color fluorescence of 488-EGFR and APC-EpCAM in MDA-MB231 was clearly observed with seven-fold plus or minus two and nine-fold plus or minus three, respectively, on the 400-nm pitch pattern of a plasmonic chip. Therefore, the 400-nm pitch contributed to the dual-color fluorescence enhancement for these wavelengths. An optimal grating pitch of a plasmonic chip improved a fluorescence image of membrane proteins with the help of the surface plasmon-enhanced field.

## 1. Introduction

Recently, plasmonics that utilize the surface plasmon resonance (SPR) technique have been widely applied in global research studies. One of these applications is biosensing [1,2,3,4,5]. Many papers in this field have already been published and the optical characteristics of SPR have been clarified in detail [6,7,8]. The electric field of SPR generates a strongly enhanced field. Various signals, such as light absorption due to molecular polarization and fluorescence emission, are then detected with high sensitivity [9,10]. Propagating SPR can provide an enhanced electric field over a wide area compared to localized SPR [11]. Among propagating SPR, the grating-coupled SPR (GC-SPR) on a metal grating surface with a 10^2^-nm scale pitch [12,13] does not require special optics such as a prism in prism-coupled SPR.

As one of the applications of GC-SPR, a plasmonic chip (a thin metal-coated substrate with a wavelength-scale grating structure) provides the enhanced fluorescence [14,15,16,17]. This occurs due to the direct coupling of incident light with the collective oscillations of free electrons (referred to as surface plasmons) on the metal surface [12,13]. For microscopic observation, it is convenient that incident light couples to surface plasmons directly, without requiring special optics such as a prism. Brighter fluorescence imaging was obtained when a plasmonic chip was used on a sample stage instead of a cover glass. Fluorescence microscopic observation using a plasmonic chip has great potential for biological application.

The surface plasmon field-enhanced fluorescence is obtained within 200 nm from the chip surface [12,13,15]. When the plasmonic chip is used for the immunosensor chip, the characteristics reduce unwanted background noise generated in the water a significant distance away from the chip surface. It is also suitable for observing the surface with a fluorescence microscope. In the resonance condition of GC-SPR, when the wavenumber of surface plasmon polaritons (spp) is equal to the sum of the wavenumber vector of incident light and the grating vector, the enhanced electric field can be obtained near the chip surface. This resonance condition is described by following Equations (1) and (2) [12].

**k**_spp_ = **k**_ph_sin *θ* ± *m***k**_g_ (1)

(2) $$\omega /c\sqrt{\varepsilon_{1}\varepsilon_{2}/\left(\varepsilon_{1}+\varepsilon_{2}\right)}=n(\omega /c)\;\sin\theta \pm m2\pi /\Lambda$$

In Equation (1), **k**_spp_, **k**_ph_, and **k**_g_ indicate the wavenumber vector of the surface plasmon, incident light, and grating, while m represents an integer. In Equation (2), *ω* and *c* indicate an angular frequency and the speed of light, and $\varepsilon_{1}$ and $\varepsilon_{2}$ are complex dielectric constants of a metal and a dielectric. *θ*, *n*, and Λ indicate the incident angle, the refractive index of the dielectric, and the pitch of the grating, respectively. According to Equations (1) and (2), the resonance wavelength of SPR shifts with a change in Λ. Under the microscope, the range of the incident angle *θ* is determined by the numerical aperture of the objective. When the resonance angle is included within the range of incident angles, an enhanced electric field is generated. The surface plasmon-enhanced effect can be obtained at both excitation and emission wavelength in fluorescence microscopy. When excitation light couples to plasmons, excitation probability increases. Moreover, when fluorescence emitted from fluorescent molecules couples again to surface plasmons, surface plasmon-coupled emission (SPCE) occurs [18].

As for the study on a breast cancer cell, the most suitable marker for a tumor-specific probe was widely examined [19]. It has been studied by several different methods including total internal reflection fluorescence microscopy (TIRFM) [20], the combination of fluorescence imaging and magnetic resonance imaging (MRI) [21], and in vivo near-infrared imaging (NIR) [22]. In our previous study, a breast cancer cell was observed with a whole array-type plasmonic chip under the fluorescence microscope [23]. The nucleus and the epithelial cell adhesion molecule (EpCAM)—a surface marker used to differentiate and classify breast cancer cells—were stained. The fluorescence image of EpCAM adsorbing to the plasmonic chip surface was enhanced more than 10 times compared with the same materials on the glass slide [23]. On the other hand, the fluorescence image of the nucleus was not enhanced because the nucleus is located a significant distant away from the surface of the plasmonic chip.

In this study, the distribution of two membrane proteins, EpCAM and epidermal growth factor receptor (EGFR), was observed in two types of living breast cancer cell lines, Michigan Cancer Foundation-7 (MCF-7) and MDA-MB-231 [24,25,26]. Dual-color fluorescence images were obtained with an epifluorescence microscope using a control coverslip and a plasmonic chip. EpCAM and EGFR positioned in a cell membrane were simultaneously detected at different fluorescence wavelengths when using different fluorescent-labeled antibodies. These antibodies are allophycocyanin (APC)-labeled anti-EpCAM antibody (emitting red fluorescence) and Alexa Fluor^®^ 488-labeled anti-EGFR antibody (emitting green fluorescence). The plasmonic chip used in this study was composed of concentric periodic circles. As such, it was referred to as a bull’s eye structure. A bull’s eye structure can utilize the light from the entire azimuth under a fluorescence microscope when coupling with surface plasmons [27,28,29,30,31,32]. To clearly detect the dual-color fluorescence even for membrane proteins with the small expression rate, the appropriate grating pitch of a bull’s eye-plasmonic chip was examined. While the 480-nm pitch can effectively couple with red light, the 400-nm pitch can create a better enhancement effect based on the resonance of the shorter wavelength region, such as green or blue light as found from Equation (1). The optimal pitch for dual fluorescence imaging of a single cell, such as the pitch contributing to the enhancement of an excited field or SPCE, was investigated on the plasmonic chip.

## 2. Materials and Methods

### 2.1. Fabrication of a Bull’s Eye-Plasmonic Chip

**Figure 1:**
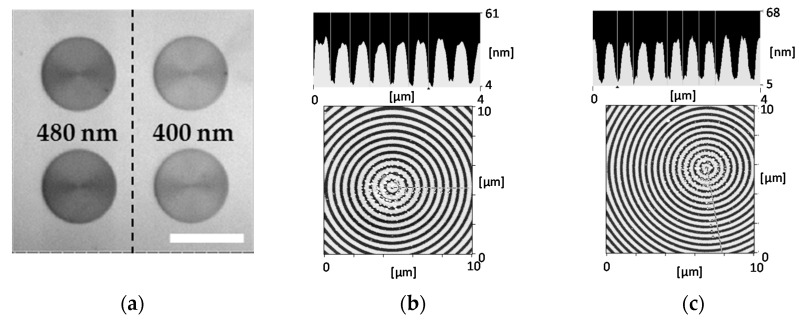
(**a**) Bright-field microscope image of arrangement for two types of 100 μm φ-bull’s eye patterns with 400 nm pitch and 480 nm pitch grating. Bar corresponds to 100 μm. Atomic force microscopy (AFM) images of a periodic structure on a bull’s eye-pattern and their contour of the cross-section images; (**b**) with 480 nm pitch, and (**c**) with 400 nm pitch.

A bull’s eye replica was fabricated by a UV nanoimprint method. A UV-curable resin (PAK-02-A; Toyo Gousei, Tokyo, Japan) was dropped on the cover glass and the mold fabricated with electron-beam lithography technique by NTT-AT (Kanagawa, Japan) was superimposed on top. The substance was exposed to UV light. In the replica, two kinds of 100 µm φ-bull’s eye patterns composed of 400 nm pitch and 480 nm pitch gratings, individually, were arranged as shown in Figure 1a. The groove depth was 30 nm for both (Figure 1b,c). The grating structure was covered with thin films of elements Ti, Ag, and Ti, and the SiO_2_ by radio frequency sputtering. Each film thickness was <1, 130 ± 20, <1, and 25 ± 5 nm, respectively. Their film thicknesses were assessed using the calibration curve of absorbance against the film forming time, which was earlier obtained by calculating the association between absorbance and the film thickness. This association was measured by fitting the SPR spectra (reflectivity against incident angle) to the simulation curve. SiO_2_ film was prepared on the surface of the plasmonic chip using the required film thickness to suppress the fluorescence quench predicted by the CPS model [33,34]. Finally, the surface of the plasmonic chip was coated with collagen film to observe the live cells. Collagen coating solution (TCC-050; Toyobo, Osaka, Japan) was dropped onto the plasmonic chip and spread by a spin coater (1000 rpm, 30 s), followed by incubating the sample for 10 min. The collagen film was finally washed with MilliQ water and the cells were then seeded. The grating pattern was evaluated by using atomic force microscopy (AFM, SPI3800N, SII) as shown in Figure 1b,c.

### 2.2. Cell Culture and Preparation for Microscopic Observation on a Glass Slide and a Plasmonic Chip

MCF-7 and MDA-MB-231 cell lines were obtained from the American Type Culture Collection. Both cell lines were cultured in Dulbecco’s modified Eagle’s medium (Gibco, Thermo Fischer Scientific, Waltham, MA, USA) containing 10% fetal bovine serum and antibiotics (100 U/mL penicillin/streptomycin (Gibco), and 250 ng/mL fungizone (Gibco)). The cell lines were then harvested using trypsin.

Alexa Fluor^®^ 488-labeled antihuman EGFR antibody (488-EGFR; Ex: 495 nm, Em: 519 nm; Biolegend, San Diego, CA, USA) and APC-labeled anti-EpCAM monoclonal antibody (APC-EpCAM; Ex: 633 nm, Em: 660 nm; Biolegend, San Diego, CA, USA) were used to stain the membranes of the membrane proteins, EGFR and CD326 (called EpCAM). EGFR is known to be highly expressed in MDA-MB-231 compared to its expression in MCF-7. EpCAM, on the other hand, is highly expressed in MCF-7 compared to its expression in MDA-MB-231. 488-EGFR and APC-EpCAM solutions were added to all cell solutions in concentrations of 1.0 × 10^8^ and 1.0 × 10^5^ molecules/cell, respectively, and they were gently mixed for 30 min in a dark place. Then, they were followed by gentle centrifugation, and the supernatant was discarded. Cell solutions were finally washed with culture medium. This process was repeated three times.

### 2.3. Microscopy

The cells were observed with an upright microscope (BX51WI; Olympus, Tokyo, Japan) using a 40× objective (UPLAN FLN ×40; Olympus, Tokyo, Japan). The light source was from an Hg lamp (BH2-RFL-T3; Olympus, Tokyo, Japan) and the detection camera was an electron multiplying charge-coupled device camera (EM-CCD, iXon; Andor, Belfast, UK). GFP (UMGFPHQ; Olympus, Tokyo, Japan) and Cy5 (Cy5-4040C; Semrock, New York, NY, USA) filter units were used for multicolor fluorescence imaging. 488-EGFR and APC-EpCAM were excited with GFP (460–480 nm) and Cy5 (605–645 nm) filters and detected with GFP (490–545 nm) and Cy5 (670–715 nm) filters, respectively. The plasmonic enhancement effect was seen in both APC-EpCAM and 488-EGFR. All fluorescence images were taken at a fixed exposure time. The EM gain for each cell and the fluorescence intensity was normalized in order to compare fluorescence images on the plasmonic chip and on the glass slides. Bright-field images were recorded without EM gain.

## 3. Results and Discussion

### 3.1. Enhanced Fluorescence Microscopic Observation of EGFR in Shorter Wavelength (Green Color)

**Figure 2:**
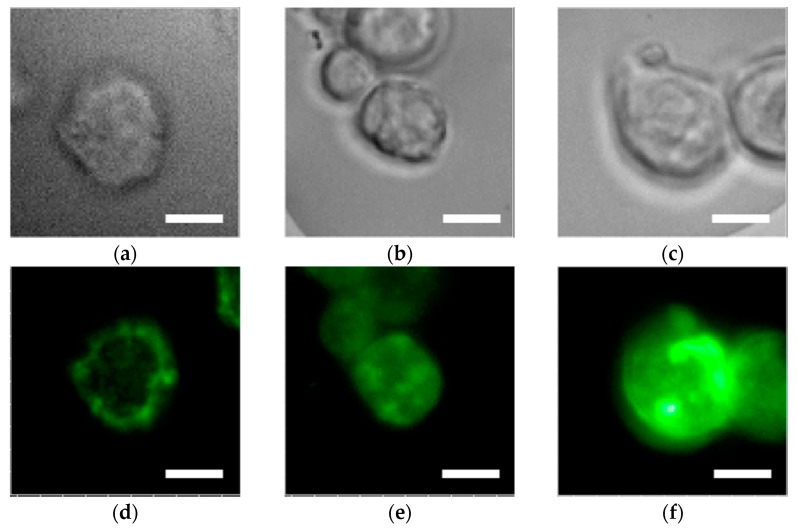
Bright-field images (**a**–**c**) and fluorescence images of 488-epidermal growth factor receptor (EGFR) (**d**–**f**) in MDA-MB-231 cells. The left, center, and right columns show images on the glass slide, and the 480-nm pitch and 400 nm pitch bull’s eye-plasmonic chips, respectively. The 488-EGFR images shown in (**d**–**f**) were adjusted to the same scale between minimum to maximum brightness corresponding to 3000 counts. Bar corresponds to 10 µm.

Bright field images (as seen in Figure 2a–c) and fluorescence images of 488-EGFR (as seen in Figure 2d–f) are shown for MDA-MB-231 cells on the glass slide (as seen in Figure 2a,d), the 480 nm pitch bull’s eye pattern (as seen in Figure 2b,e), and the 400 nm pitch bull’s eye pattern (as seen in Figure 2c,f). The darkest fluorescence image was seen on the glass slide, in which the largest fluorescence intensity was detected at the edge of the cell (Figure 2d). The cell outline was clearly observed due to the integration of fluorescence in the cell membrane along the *z*-axis.

In contrast, on the 400-nm pitch bull’s eye plasmonic chip, the brightest fluorescence was detected among the chips [18]. Fluorescence intensity was especially enhanced at the cell membrane adsorbing to the plasmonic chip as shown in Figure 2f. The enhancement factor was evaluated as follows. Background noise including direct and scattering excited light was subtracted from fluorescence intensities while the mean fluorescence intensities on the glass slide and the plasmonic chip were evaluated. The enhancement factor was obtained as the ratio between the mean value on a plasmonic chip and the mean value on a glass slide. To evaluate the enhancement factor of the fluorescence at the midpoint of the cells, three to five cells were chosen on a glass slide and a plasmonic chip, individually. A three-fold plus or minus one increase of fluorescence intensity was found at the center area of a cell on the 480-nm pitch bull’s eye-plasmonic chip.

The surface plasmon-enhanced electric field exponentially decreased with the distance from the metal. Therefore, it was discovered that enhanced fluorescence is obtained from the cells adsorbing on the collagen surface. Moreover, on the 400-nm pitch bull’s eye-plasmonic chip, fluorescence intensity at the cell’s midpoint was strongly enhanced compared to that on the 480-nm pitch bull’s eye-plasmonic chip. The enhancement factor was seven plus or minus two. The surface plasmon enhancement effect was found to depend on the pitch of the grating and the wavelengths of excitation and emission. At the direction of substrate normal, the resonance wavelengths were calculated from Equation (1) as 570 nm and 670 nm at the 400-nm pitch and 480 nm pitch, respectively. Therefore, the 400-nm pitch, rather than the 480-nm pitch, is appropriate to collect the SPCE effectively through the objective at the emission wavelength ranges of 488-EGFR emission.

**Figure 3:**
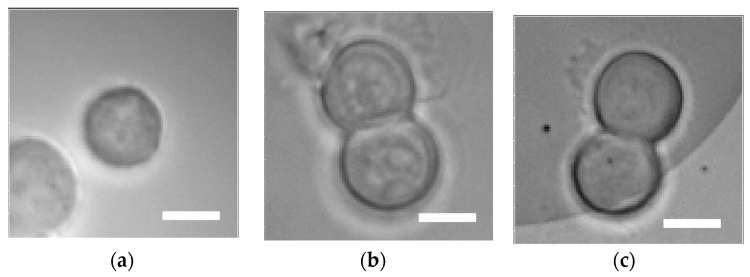

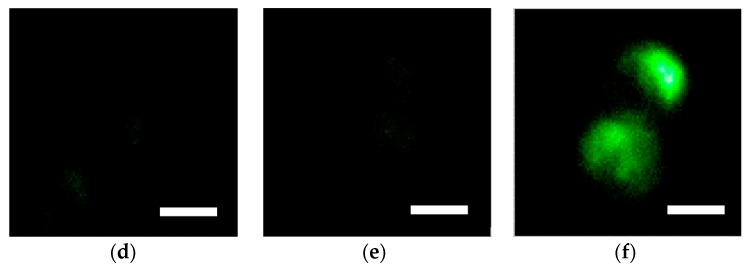
Bright-field images (**a**–**c**) and fluorescence images of 488-EGFR (**d**–**f**) in MCF-7 cells. The left, center, and right columns show images on the glass slide, the 480-nm pitch, and the 400-nm pitch bull’s eye-plasmonic chip, respectively. The 488-EGFR images shown in (**d**–**f**) were adjusted to the same scale between minimum to maximum brightness corresponding to 3000 counts. Bar corresponds to 10 µm.

The fluorescence microscopic observation of 488-EGFR in MCF-7 was also conducted in the same manner as for MDA-MB-231. Fluorescence images were compared between those on the glass slide (as seen in Figure 3a,d) and those on the 480-nm pitch (as seen in Figure 3b,e) and the 400-nm pitch (as seen in Figure 3c,f). On the glass slide, fluorescence intensity was too weak to evaluate the distribution of the EGFR (Figure 3d). In contrast, the 400-nm pitch provided a brighter fluorescence image (as seen in Figure 3f) than that for 480 nm pitch (as seen in Figure 3e). Despite the small expression amount of EGFR in the MCF-7 cell [35], the distribution was clearly visible on the plasmonic chip. While the enhanced electric field for silver is beneficially larger for longer wavelengths such as a red light, the enhancement factor for 488-EGFR was seven plus or minus two in both MDA-MB231 and MCF-7 cells likely due to the complex dielectric constants of silver. The 400-nm pitch bull’s eye plasmonic chip precisely detected membrane proteins with a low expression rate under the fluorescence microscope with green emission.

### 3.2. Dual-Color Fluorescence Microscopic Observation in MDA-MB-231

In our previous study [23], surface plasmon-enhanced dual-color fluorescence observation of breast cancer cells was performed for membrane protein (EpCAM) and cell nucleus stained with APC-anti EpCAM antibody (APC-EpCAM) and 4’, 6-diamino-2-phenylindole, respectively. Only the fluorescence emitted from APC-EpCAM within the surface plasmon-enhanced field, i.e., within the distance of 200 nm from the metal surface, was enhanced. In this study, two kinds of membrane proteins (EGFR and EpCAM) in MDA-MB-231 cells were stained with two kinds of fluorescently labeled antibody (488-EGFR and APC-EpCAM).

**Figure 4:**
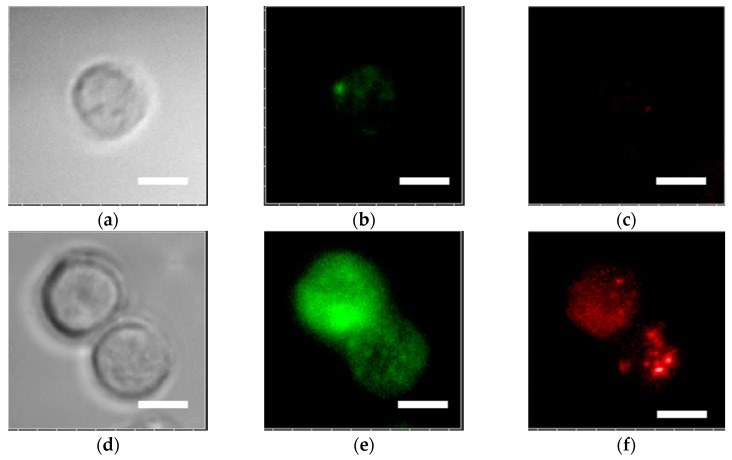
Bright-field images (**a**,**d**), fluorescence images of 488-EGFR (**b**,**e**), and fluorescence images of allophycocyanin-labeled anti-epithelial cell adhesion molecule antibody (APC-EpCAM), (**c**,**f**) in MDA-MB-231 cells. The upper and lower columns show images on the glass slide and the 400-nm pitch bull’s eye-plasmonic chip, respectively. The 488-EGFR and APC-EpCAM images shown in (**b**,**e**) or (**c**,**f**) were adjusted to the same scales between minimum and maximum brightness corresponding to 3000 and 2000 counts, respectively. Bar corresponds to 10 µm.

Fluorescence images of 488-EFGR on the 400-nm pitch were brighter than those observed on the glass slide (Figure 4b,e). The enhancement factor of 488-EGFR on the 400-nm pitch was seven plus or minus two as described in Section 3.1. In turn, fluorescence of APC-EpCAM was also enhanced on the 400-nm pitch bull’s eye. This is considered to be due to the fact that the range of excitation wavelength is closed to the resonance wavelength when observing it at the substrate normal for a 400-nm pitch. The relative expression rates of EpCAM were found to be 0.26 and 0.0009 for MCF7 and MDA-MB231, respectively [36]. Regardless of the low expression rate of EpCAM in MDA-MB-231, the distribution of EpCAM was clearly observed as shown in Figure 4f with the enhancement factor at nine plus or minus three.

Surface plasmon-enhanced fluorescence intensity depended on the wavelength, and was generally larger at a longer wavelength of light. Therefore, APC-EpCAM with emission at a longer wavelength was enhanced more strongly than 488-EGFR. This enhancement effect was improved on the 480-nm pitch by up to 10-fold (not shown here). This is due to 480-nm pitches making use of the SPR state for the wavelength ranges of excitation (605–645 nm) and emission (670–715 nm) when compared to 400 nm pitches. However, when the expression distributions of both 488-EGFR and APC-EpCAM were simultaneously observed with dual-color fluorescence, the 400-nm pitch was superior to the 480-nm pitch. The surface plasmon coupling at the 400-nm pitch contributed to the enhancement of the excitation field for APC-EpCAM and to the collection of the SPCE for 488-EGFR effectively under the microscope.

It is generally complex when dual-color fluorescence intensities are simultaneously enhanced based on plasmonic resonance conditions. An example of dual fluorescence imaging is when surviving dual fluorescence resonance energy transfers molecular beacons with fluorophores to form one donor–acceptor pair. This occurs in order to improve the specific detection of cancer cells [37]. In this study, the 400-nm pitch of a plasmonic chip exhibited an advantage for the dual-color fluorescence microscopic observation. The existence of two kinds of proteins EGFR and EpCAM and their distribution were clearly demonstrated by dual-color fluorescence imaging. As shown in Figure 4b,c, it is difficult to determine the distribution of EGFR and EpCAM on the glass slide. On the plasmonic chip, as shown in the cell for a bottom side in Figure 4e,f, the 488-EGFR homogeneously dispersed but APC-EpCAM concentrated to the bottom right. The different distributions for two kinds of proteins were demonstrated with the 400-nm pitch plasmonic chip.

In the future, integration of nanoparticles on the plasmonic chip and combination between the plasmonic chip and confocal microscopy [38] may improve the fluorescence imaging. The former may improve the detection sensitivity by combining the enhanced electric field between GC-SPR and localized SPR while the latter can improve the spatial resolution in fluorescence images. The plasmonic chip is a prospective candidate for developing molecular sensing and microscopic imaging.

## 4. Conclusions

In this study, dual-color fluorescence observations for two kinds of membrane proteins, EGFR and EpCAM, were detected with a plasmonic chip by using surface plasmon-enhanced fluorescence microscopy in two kinds of breast cancer cell lines, MDA-MB-231 and MCF-7. The enhancement factors of 488-EGFR and APC-EpCAM were seven plus or minus two and nine plus or minus three, respectively, in the 400-nm pitch plasmonic chip. Fluorescence intensity on the 400-nm pitch was sufficient to observe expression distribution of the membrane proteins. The 400-nm pitch was advantageous for dual-color fluorescence (green and red) observation.

This enhancement effect will enable the real-time fluorescence detection of a single-nanoparticle. By selecting an appropriate grating pitch, the surface plasmon-enhanced fluorescence effect allows multicolor fluorescence microscopic observation. This offers a great advantage for medical fields, such as in early-stage diagnosis.
